# Pathways for mitigating thermal losses in solar photovoltaics

**DOI:** 10.1038/s41598-018-31257-0

**Published:** 2018-09-03

**Authors:** Rodolphe Vaillon, Olivier Dupré, Raúl Bayoán Cal, Marc Calaf

**Affiliations:** 1 Univ Lyon, CNRS, INSA-Lyon, Université Claude Bernard Lyon 1, CETHIL UMR5008, F-69621 Villeurbanne, France; 2 Department of Mechanical Engineering, University of Utah, Salt Lake City, UT 84112 USA; 3 Instituto de Energía Solar, Universidad Politécnica de Madrid, 28040 Madrid, Spain; 4 Ecole Polytechnique Fédérale de Lausanne (EPFL), Institute of Microengineering (IMT), Photovoltaics and Thin-Film Electronics Laboratory, Rue de la Maladière 71b, 2002 Neuchâtel, Switzerland; 5 Department of Mechanical and Materials Engineering, Portland State University, Portland, OR 97207 USA

## Abstract

To improve the performance of solar photovoltaic devices one should mitigate three types of losses: optical, electrical and thermal. However, further reducing the optical and electrical losses in modern photovoltaic devices is becoming increasingly costly. Therefore, there is a rising interest in minimizing the thermal losses. These correspond to the reduction in electrical power output resultant of working at temperatures above 25 °C and the associated accelerated aging. Here, we quantify the impact of all possible strategies to mitigate thermal losses in the case of the mainstream crystalline silicon technology. Results indicate that ensuring a minimum level of conductive/convective cooling capabilities is essential. We show that sub-bandgap reflection and radiative cooling are strategies worth pursuing and recommend further field testing in real-time operating conditions. The general method we propose is suitable for every photovoltaic technology to guide the research focused on reducing thermal losses.

## Introduction

The well known chart of best research-cell efficiencies regularly issued by the National Renewable Energy Laboratory illustrates decades of research and engineering for designing solar cells with ever growing performances^1^. In this chart, efficiencies are rated in the so-called Standard Test Conditions (STC), *i.e*. for the one Sun (AM1.5) illumination and a cell at a temperature of 25 °C. Unfortunately, STC are rarely met in the field and most solar photovoltaic installations are operating at temperatures greater than 25 °C. More importantly, the efficiency of the vast majority of photovoltaic converters drops when temperature increases, with a rate commonly comprised between −0.1 and −0.5% K^−1^ ^2^. Because of the substantial effect of these thermal losses on the energy yield^3^ and production potential in the world^4^, there is an imperative need for mitigating them.

**Figure 1:**
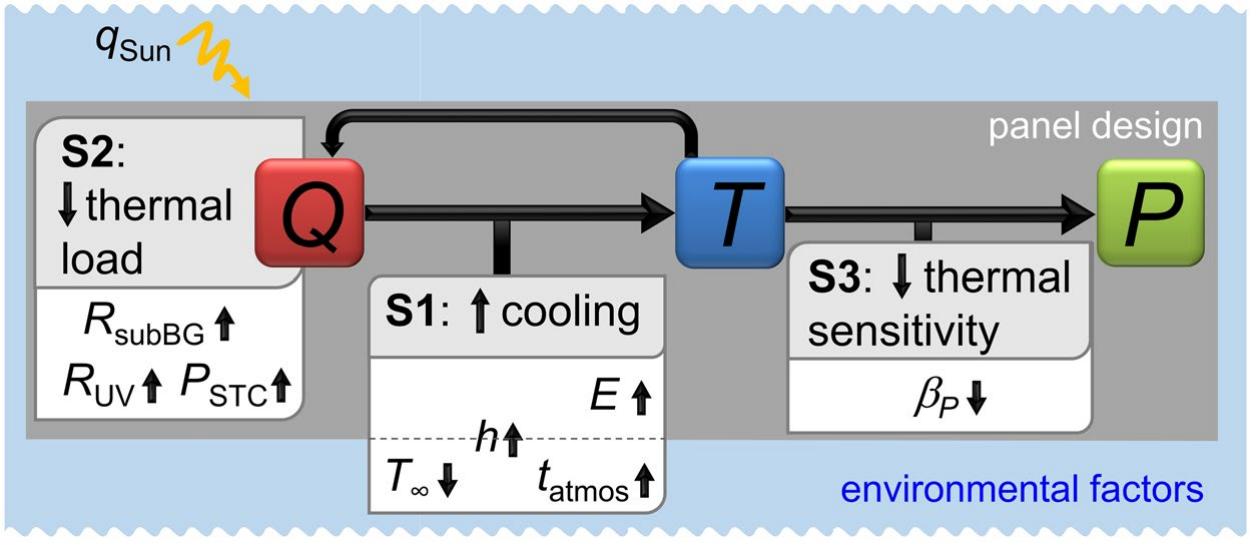
The three strategies for mitigating the thermal losses: (S1) maximizing cooling, (S2) minimizing thermal load, (S3) minimizing thermal sensitivity.

**Figure 2:**
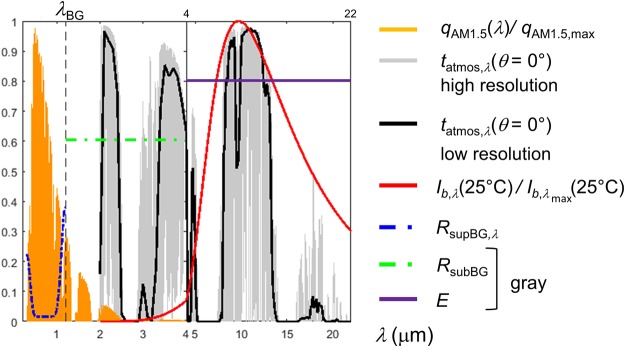
Normalized AM1.5 solar spectrum, high and low resolution clear sky normal (*θ* = 0) atmospheric transmissivity, normalized blackbody intensity at 25 °C, solar panel sup-bandgap reflectance (*R*_*supBG*_, *λ* ≤ *λ*_*BG*_), model gray sub-bandgap reflectance (*R*_*subBG*_, *λ*_*BG*_ < *λ* ≤ 4*μ*m) and emittance (*E*, 4 *μ*m < *λ *≤ 22 *μ*m).

Three strategies are available^2^ (Fig. 1). The first option (S1) is to maximize cooling, by conduction/convection with a colder medium, and by radiation towards the surroundings and the cold outer space under clear sky conditions. The second option (S2) is to minimize the thermal load (internal heat source, *Q*) in the panel. The aim of these first two strategies is to prevent the panel temperature (*T*) from rising too far above the outdoor temperature (*T*_∞_). The last option (S3) is to minimize the thermal sensitivity (temperature coefficient *β*_*P*_) of the electrical power output (*P*). Efficiency of these three strategies depends primarily on environmental conditions (Figs 1 and 2) and design. Environmental conditions are solar irradiation flux (*q*_*Sun*_), outdoor temperature, wind velocity, and clear sky atmospheric transmissivity (*t*_*atmos*_), which depend on where the solar photovoltaic panels are installed. Unfortunately, these conditions can rarely be manipulated to improve the efficiency of the solar PV systems. However, in terms of design, several opportunities for mitigating the thermal losses exist. For example, to maximize cooling, panels can be engineered to increase the heat transfer coefficient (*h*) and emittance (*E*). To minimize the thermal load, sub-bandgap reflectance (*R*_*subBG*_) can also be increased to avoid absorbing photons that are useless for photoconversion (*i.e*. incident photons with energy lower than the bandgap). Additionally, the very high energy photons provide a surplus of energy above the bandgap which is converted into heat by thermalization. Filtering out these photons by increasing the reflectance in the ultra-violet range (*R*_*UV*_) can also be beneficial in certain configurations. Further, a fact that is often overlooked is that any increase in electrical power produced in STC (*P*_*STC*_) comes with less heat generated in the device. This means that devices with higher efficiencies naturally operate at lower temperatures than their low efficiency counterparts. Last but not least, minimizing the temperature coefficients (*i.e*. the dependence of power losses on temperature, *β*_*P*_) would directly help lower the thermal losses. These different strategies have only recently started to be investigated^2,5–13^.

Here, we reveal with quantified gains in efficiency the relative benefits of the different general strategies for mitigating the thermal losses in the case of state-of-the-art crystalline silicon panels. In particular, we show that ensuring a minimum level of conductive/convective cooling capabilities is essential, motivating further research on panel designs and field arrangement. Additionally, we show that sub-bandgap reflection and radiative cooling are strategies worth pursuing since, for example, they can boost by several percents relative conversion efficiency of current industrial passivated emitter and rear cells (PERC) operating in realistic conditions. As a result of this comprehensive analysis, we highlight the optimum strategies for mitigating the thermal losses and thus increasing the energy yield of the next generation photovoltaic installations as a function of their operating conditions.

## Impacts of thermal loss mitigation strategies for crystalline silicon

Currently, the dominant technology on the market is crystalline silicon cells, hence their corresponding characteristics are selected for our study case. In this regard, realistic sup-bandgap reflectance (*R*_*supBG*_) and external quantum efficiency (*EQE*), both required by the simulations, are extracted for an industrial grade PERC cell from^14^. In this case, efficiency in STC is 20.69% (*P*_*STC*_ = 206.9 W m^−2^). This power value in STC conditions is used in the following analysis to rate the impact of thermal losses on the electrical power output. Unless specified otherwise, the temperature coefficient (*β*_*P*_) is set to −0.45% K^−1^, the standard value for crystalline silicon cells. Also for scaling convenience, a −10% STC rated power change is equivalent to +22.2 °C panel operating temperature and −2.07% absolute conversion efficiency changes.

**Figure 3:**
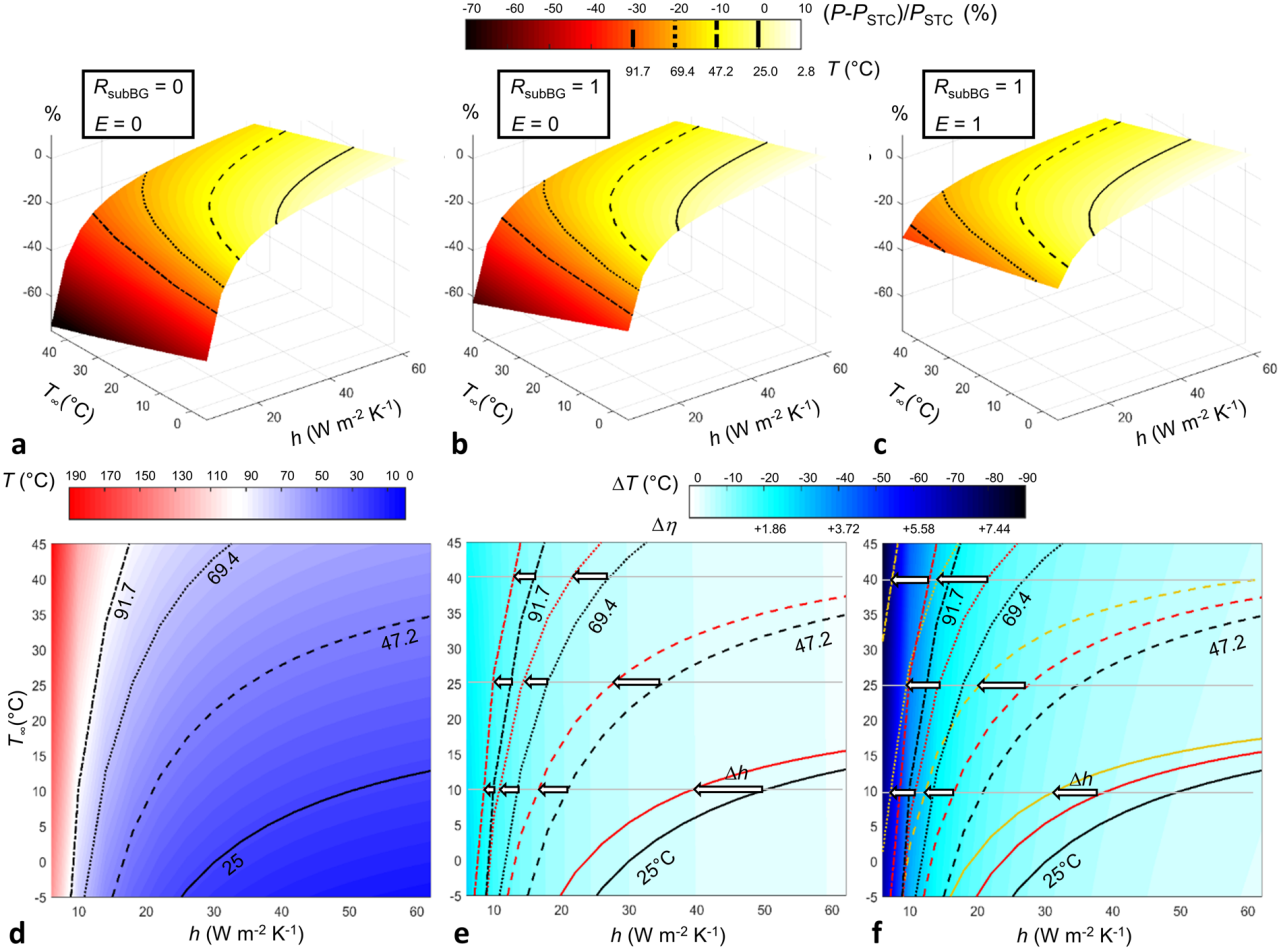
(**a**–**c**) Electrical power loss to that in STC (%), as a function of the conductive/convective heat transfer coefficient (*h*) and outdoor temperature (*T*_∞_). The 0, −10, −20, −30% loss isolines (corresponding to panel temperatures of 25, 47.2, 69.6 and 91.7 °C) are depicted. (**a** and **d**) Base case, no sub-bandgap reflection (*R*_*subBG*_ = 0), no radiative cooling above 4 *μ*m (*E* = 0). (**b** and **e**): case with maximum sub-bandgap reflection (*R*_*subBG*_ = 1) and no radiative cooling above 4 *μ*m (*E* = 0). (**c** and **f**) case with maximum sub-bandgap reflection (*R*_*subBG*_ = 1) and maximum radiative cooling above 4 *μ*m (*E* = 1). (d) panel temperature as a function of *h* and *T*_∞_. Lines represent isotherms at 25, 47.2, 69.6 and 91.7 °C. (e and f) panel temperature drop with respect to the base case (**d**) as a function of *h* and *T*_∞_. A scale of the corresponding absolute gain in efficiency (Δ*η*) is added. Color lines represent isotherms at 25, 47.2, 69.6 and 91.7 °C (black lines: base case). Arrows indicate the decrease in conductive/convective cooling (*h*) required for reaching a given panel temperature for specific outdoor temperatures. The temperature coefficient (*β*_*P*_) is set to −0.45% K^−1^. Ensuring a minimum level of conductive/convective cooling capabilities is essential. Benefits of minimizing the thermal load by sub-bandgap reflection (S2) and of maximizing cooling by thermal radiation toward the cold outer space (S1) are undeniable and quantifiable, as a function of operating conditions.

In a first step, with the aim of determining the optimum strategies for mitigating the thermal losses, a parametric analysis is performed with model cases involving spectrally constant - gray - (Fig. 2) sub-bandgap reflectance (*R*_*subBG*_, contributing to S2) and emittance (*E*, contributing to S1). Figure 3a,b and c display the drop in electrical power output as a function of heat transfer coefficient (*h*) and outdoor temperature (*T*_∞_, contributing to S1), in the cases where there is neither sub-bandgap reflection nor radiative cooling (*R*_*subBG*_ = 0 and *E* = 0, base case), sub-bandgap reflection is maximum without any radiative cooling (*R*_*subBG*_ = 1 and *E* = 0), and both sub-bandgap reflection and radiative cooling are maximum (*R*_*subBG*_ = 1 and *E* = 1), respectively. Since the power drop is rated with respect to power in STC, a power isoline corresponds to a specific panel temperature. In the base case, Fig. 3d provides a contour map of panel temperature, where the 25, 47.2, 69.4 and 91.7 °C isotherms are highlighted. These lines serve as reference in the following of this work. The base case indicates only the effect of conductive/convective cooling on electrical power as a function of outdoor temperature. Consistently, conditions in which the panel temperature is 25 °C (*T*_*STC*_) or below are very limited (*T*_∞_ < 10 °C and *h* > 25 W m^−2^ K^−1^). Therefore, it is worth noting that increasing convective cooling is significantly beneficial in the adverse thermal conditions up until reaching a plateau. As expected, the rate of convective cooling improvement is diminished when the outdoor temperature is rising. Since the base case is thermally inefficient compared to actual - more realistic - panels analyzed in the next subsection, the extreme temperatures calculated in the worst operating conditions may seem a bit excessive (~190 °C).

When sub-bandgap reflection is added at its fullest to mitigate the thermal losses (S2), a temperature drop is observed in Fig. 3e. The impact of lowering the thermal load is the largest when the convective cooling is the weakest with a very small dependence on outdoor temperature. Isotherms are superimposed in Fig. 3e to indicate with arrows the diminution in required convective cooling (Δ*h*) for the panel temperature to reach a specified value when a maximum sub-bandgap reflection is added. For example, since the drop in heat load caused by sub-bandgap reflection is constant, for a given outdoor temperature the smaller the isotherm value (thus *T*−*T*_∞_) is, the larger the diminution in convective cooling will be. Equivalently, for a given isotherm, a higher outdoor temperature will result in a larger diminution. The scale in conversion efficiency absolute gain (Δ*η*), which has a correspondence with that of temperature drop (+0.093%/−1 °C), provides a quantified benefit of reflecting the photons with sub-bandgap energy.

When maximum radiative cooling, contributing to S1, is added (Fig. 3c and f), similar general trends are observed. The impact of radiative cooling grows as convective cooling becomes less efficient, and amplifies with an increase in outdoor temperature (white arrows in Fig. 3f). The difference between the largest (658.5 W m^−2^) and smallest (569.0 W m^−2^) heat load (*Q*) is 89.5 W m^−2^, almost 9% of the incoming solar irradiation flux (1000.6 W m^−2^). Depending on outdoor temperature and the heat transfer coefficient, the relative contribution of radiative cooling to total cooling varies, and is never negligible (the cooling radiative heat flux *q*_*rad*_ is comprised between 80.0 and 318.2 W m^−2^). Therefore, a gain is always observed when adding radiative cooling, in particular in conditions where convective cooling tends to become inefficient. This gain is particularly important in the worst operating conditions, hence leading to more realistic temperatures than the base case (see Fig. 3d and f).

On the contrary, filtering out the high energy photons is never found to be beneficial except in the base case, with a low heat transfer coefficient (<10 W m^−2^ K^−1^) and a temperature coefficient magnitude greater than 0.35%.

**Figure 4:**
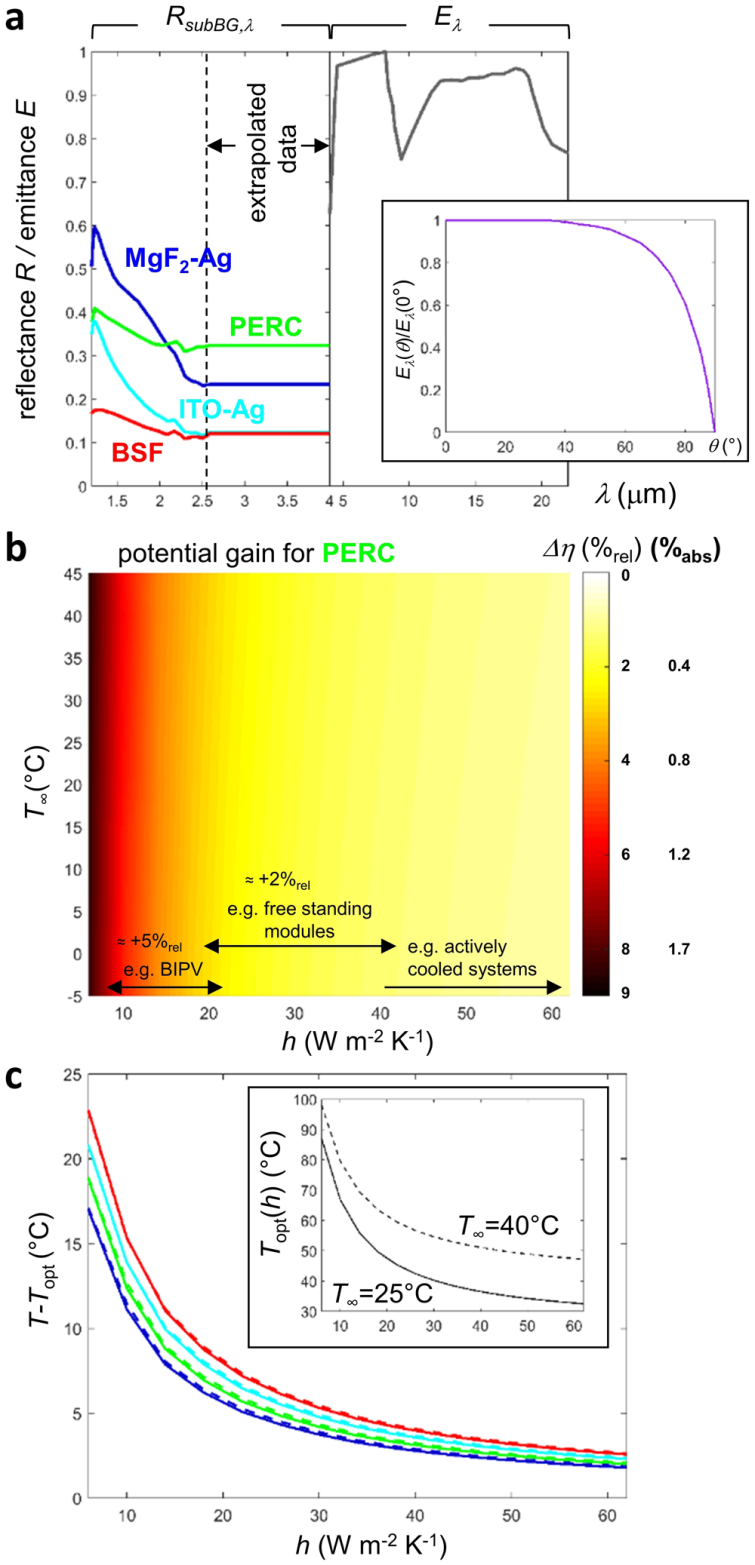
Case with realistic radiative properties (*λ* > *λ*_*BG*_). (**a**) Selected spectral sub-bandgap reflection (*R*_*subBG*,*λ*_, for MgF_2_-Ag, PERC, ITO-Ag and Al-BSF back reflectors) and emittance above 4 *μ*m (*E*_*λ*_, soda-lime glass). Insert: weighting factor applied to the emittance to account for its dependence on polar angle *θ* (*E*_*λ*_(*θ*)/*E*_*λ*_(0)). (**b**) Relative and absolute efficiency gains (%) by reaching the optimum case with maximum sub-bandgap reflection and radiative cooling above 4 *μ*m (*R*_*subBG*,*λ*_ = 1 and *E*_*λ*_ = 1, see Fig. 3c and f), in the case of the PERC back reflector, as a function of the conductive/convective heat transfer coefficient (*h*) and outdoor temperature (*T*_∞_). (**c**) Temperature difference with that of the optimum case for the four back reflectors. There is still some room for improving sub-bandgap reflection and radiative cooling beyond the state-of-the-art.

In a second step, in order to assess the current state-of-the-art technologies and foreseeable progress beyond, realistic sub-bandgap reflectance (*R*_*subBG*_) and emittance (*E*) are considered (Fig. 4a). Four back reflectors (MgF _2_-Ag, PERC, ITO-Ag and Al-BSF) are selected using data from^15^, extrapolated beyond 2.5 *μ*m up to 4 *μ*m (even though this range contributes less than 1% of the total AM1.5 irradiation power). The selected reflectance data for the Al-BSF back reflector are consistent with those provided in^16^. Above 4 *μ*m, panel emittance is dominated by that of glass^16^, hence the soda-lime glass emittance spectrum given in^12^ is applied. Dependence of emittance with polar angle is modelled using a weighting factor inferred from^8^ for every wavelength. The radiative cooling (at *T* = 25 °C) is not that of a blackbody isotropically transmitted through the atmosphere. Instead it is affected by the decline of atmospheric transmissivity with polar angle (−13%), by soda-lime glass real spectral emittance (additional −13%) and in a lesser extent by the decline of glass emittance with polar angle (additional −5%). This is presented in Table 1.

**Table 1:** Radiative cooling heat flux (*q*_*rad*_,W m^−2^) at 25 °C for various atmosphere transmissivity and panel emittance configurations.

*t*_*atmos*,*λ*_(*θ*)	*E*_*λ*_(*θ*)		*q*_*rad*_(25 °C)
isotropic	blackbody	isotropic	131.7
non isotropic			116.8
	soda-lime glass		101.7
		non isotropic	96.6

Figure 4b illustrates how much power rated with the power output in STC would be gained by engineering the panel with the PERC cells (back reflectors) in a way it would have properties of the optimum case with maximum sub-bandgap reflection and radiative cooling (*R*_*subBG*,*λ*_ = 1 and *E*_*λ*_ = 1, see Fig. 3c and f). Consistent with the results in the previous section, improvements induced by the addition of sub-bandgap reflectance (S1) and radiative cooling (S2) would be the largest when conductive/convective cooling (S2) is the lowest. These improvements are almost independent from outdoor temperature. This kind of operating condition can be encountered in certain PV applications such as BIPV (Building Integrated PV) or space. In BIPV, the modules are used as roofing or facade elements and the convection at the rear of the module can be reduced and sometimes even completely suppressed. In space applications, the modules are surrounded by vacuum and the convection is thus null. Figure 4b illustrates that thermal engineering has the potential of boosting conversion efficiencies by more than 5%_*rel*_. Even for standard free-standing modules, the common configuration for large scale PV plants, efficiencies could be increased by about 2%_*rel*_. The exact value depends on installation site (in particular the average wind speed and orientation) and on certain module characteristics. Figure 4c shows how much hotter is the panel in comparison to the optimum case, for the different back reflector technologies. For example, when *T*_∞_ = 25 °C and *h* = 18 W m^−2^ K^−1^, the panel temperature is 6.2, 6.9, 7.9 and 8.8 °C larger than the optimum one for MgF_2_-Ag, PERC, ITO-Ag and Al-BSF reflectors, respectively. This result highlights the already ongoing improvement of PV devices thermal properties (which is a beneficial side-effect of optical and electrical optimizations) and the potential for further amelioration. When conductive/convective cooling is large, there are only few degrees to be gained, almost independently from outdoor temperature. The interest of reducing sub-bandgap heat load and improving radiative cooling grows as conductive/convective cooling is lowered. The reductions in operating temperature shown in Fig. 4c translate directly in improved efficiencies and energy yields via the temperature coefficients of the devices. In the example of PERC cells, the enhancements would lead to a conversion efficiency gain of about 2%_*rel*_ (see also Fig. 4b).

## Discussion

Among the multiple strategies for mitigating the thermal losses, conductive/convective exchange with a cooler medium should be one of the primary options to pursue given the strong non-linear behavior of the solar panels’ temperature with the convective heat transfer coefficient as illustrated in Figs 3 and 4, providing an important improvement in efficiency. While part of it is out of direct control because it depends on outdoor weather conditions (wind velocity and outdoor temperature), results show that changes in the convective heat transfer coefficient, even by a small amount, can lead to substantial benefits. This observation indicates that ensuring a minimum level of conductive/convective cooling capabilities is required, and hence it motivates further research on engineering panel designs and field arrangements using numerical simulations (e.g.^17,18^), as well as scaled and field experiments to derive approaches that enhance passive convection.

Additionally, calculations in the model cases also show the importance to having a certain contribution of sub-bandgap reflection and radiative cooling, since it leads to gains in absolute efficiency on the order of a few percents. Reflecting the photons useless for photoconversion lowers the thermal load, with the largest benefits happening when conductive/convective cooling is weak. The sub-bandgap potential heat load reduction and its impacts obviously depend on the bandgap of the semiconductor used^11^. For a given technology, *i.e*. crystalline silicon cells, the cell back reflector plays a relevant role^9^. Simulation results (Fig. 4b,c) demonstrate room for improvements beyond the case of PERC cells. Calculations of mid-infrared emittance of crystalline silicon solar cells reveal other design options for minimizing sub-bandgap absorption, especially by tuning the texture steepness of the front and rear surfaces of the cell. However, this procedure may also lead to possible degradation of absorption in the sup-bandgap spectral region^16^. Adding a smartly designed photonic structure on top of the encapsulated cells is another option for improvement^10,13^, but for practical implementation cost still remains an issue.

In clear sky conditions, radiative cooling is always advantageous, especially in environmental conditions with high outdoor temperatures and/or small heat transfer coefficients. This is because radiation heat flux is a non-linear function of the panel temperature, regardless of outdoor temperature, while conductive/convective cooling depends on the difference between panel and outdoor temperature. As a result, it is consistent to find that the ratio of radiative to total cooling heat flux is maximum with high outdoor temperatures and small heat transfer coefficients. Any additional radiative cooling comes with a decrease of conductive/convective cooling^8^ because the heat load is barely varying yet the total cooling is always increased through radiative cooling. Current photovoltaic technologies using low-iron glass cover-sheets are already performing quite well^2^ [and citations therein] in terms of radiative cooling. However, additional gains are still possible. Table 1 establishes a hierarchy between preventing glass emittance drop at large angles^8^ and making the panel a blackbody emitter, for example by using photonic structures^10,12,19^. Further, the drop of atmospheric transmissivity at grazing angles should not be omitted, and the effect of local composition of the atmosphere^12^ should also be considered. Sub-bandgap heat load mitigation and radiative cooling performances are varying under low and high solar illumination conditions^11^. Similarly, if high-energy photon filtering is essentially inefficient under one Sun terrestrial illumination, it might be otherwise in concentrated and space photovoltaics.

**Figure 5:**
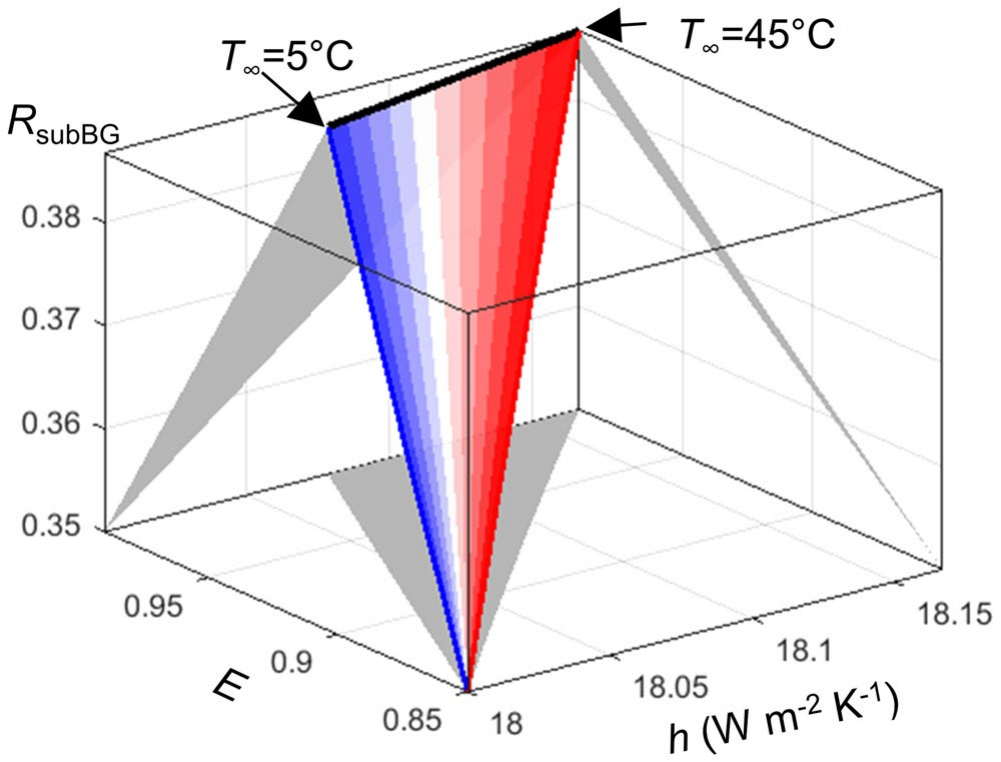
Streamlines of maximum gradient of electrical power output, when the conductive/convective heat transfer coefficient *h* is 18 W m^−2^ K^−1^, panel emittance *E* is gray and equal to 0.85, sub-bandgap reflectance *R*_*subBG*_ is gray and equal to 0.35, and for a set of outdoor temperatures (*T*_∞_) ranging from 5 °C (blue) to 45 °C (red). Projections on coordinate planes are guides to assess the separate impact of couples of parameters. Such streamlines of maximum gradients indicate which strategy would be the most beneficial for mitigating thermal losses, as a function of actual panel characteristics (emittance and sub-bandgap reflectance) and/or environmental factors (heat transfer coefficient, outdoor temperature).

In the interest of determining the optimal strategies for mitigating thermal losses in solar phototvoltaics and hence help improve the state-of-the-art photovoltaic devices, results illustrate that enhancing radiative and conductive/convective cooling (S1) or reducing the thermal load (S2) by reflecting photons with sub-bandgap energies are very efficient options that should be pursued. Figure 5 depicts streamlines leading to maximum gains in electrical power output assuming an initial state corresponding to a standard panel in average atmospheric conditions. Specifically, the initial state corresponds to a panel with gray emittance and sub-bandgap reflectance (similar to the realistic soda-lime glass cover and PERC cells, *E* = 0.85, *R*_*subBG*_ = 0.35, see the previous section), with a heat transfer coefficient corresponding to typical average wind velocities (*h* = 18 W m^−2^ K^−1^), and for a set of outdoor temperatures ranging between 5 °C and 45 °C.

Streamlines indicate the worthiness of increasing the emittance up to unity, specially in low outdoor temperature conditions. When the outdoor temperatures are high, the blackbody radiation flux is large, and hence it diminishes the effect of improving the emittance. A similar trend is observed for the sub-bandgap reflection, with about 10% increase in sub-bandgap reflectance. It is worth noting that if emittance and sub-bandgap reflection enhancements are of the order of a dozen percents, the streamlines of maximum gradient involve increases of the heat transfer coefficient of only less than a percent. This highlights the fact that increasing passive conductive/convective cooling by only a few percents produces significant changes in the efficiency of the system, thus emphasizing the research opportunity that this pathway represents. Note that this analysis is subject to the environmental conditions relative to a site and the properties of the solar panel that one would like to use. Therefore, this analysis should be redone for each specific study site and conditions.

It should not be forgotten that any increase of conversion efficiency in STC comes with a heat load mitigation (S2), thus with less thermal losses. For example, in the case where sub-bandgap reflection and radiative cooling are maximum (*R*_*subBG*_ = 1, *E* = 1), additional simulations indicate that a 1% increase of efficiency in STC leads to conversion efficiency absolute gains comprised between 0.73 to 1.11%, depending on operating conditions.

Hot carrier cells, up- and down- conversion are other strategies available for lowering the heat load^2^. However these options are still ongoing research topics, thus not applicable yet. Their impact on mitigating the thermal losses would be similar to those from sub-bandgap reflection, with a larger potential for reducing the heat source.

Evidently, the most radical approach for mitigating thermal losses is to engineer cells with a lower temperature sensitivity of power output (S3). For example, if instead of the common value of −0.45% K^−1^ the minimum temperature coefficient −0.238% K^−1^ accounting for radiative and Auger recombination mechanisms^2^ is applied, then for the same conversion efficiency in STC (20.69%), a −10% STC rated power change would happen for a much larger panel temperature rise (+42 °C instead of 22.2 °C) in Fig. 3. Concurrently, temperature drops resulting from cooling (S1) and thermal load reduction (S2) strategies would induce smaller conversion efficiency gains. Minimizing the temperature coefficient of solar cells is definitely worth pursuing, by capitalizing on a fine knowledge of the physics ruling variations of optical and electrical losses with temperature^20^ for different technologies^2^.

In summary, the proposed assessment of pathways for mitigating the thermal losses in the case of crystalline silicon solar photovoltaic panels indicates that sub-bandgap reflection (S2) and when possible radiative cooling (S1) are important strategies to pursue. Further, because there is still room for improvement beyond the current state-of-the-art technology, engineering panel designs with better conductive/convective cooling capacities is also shown to lead to important improvements in efficiency. Evidently, efficient ways for mitigating thermal losses are to increase power output in STC (S2) and more importantly, to decrease temperature sensitivity (S3). Our analysis of the pathways for mitigating thermal losses in solar photovoltaics strongly supports testing of their multiple possible implementations, for multiple technologies and in real time-varying operating conditions, as done very recently in^21,22^.

## Methods

The AM1.5 spectrum^23^ is selected for the incident solar irradiation (*q*_*Sun*_). It is defined between 0.28 and 4 *μ*m, with a contribution of wavelengths larger than 2.5 *μ*m, which is less than 1% of the total incident power (1000.4 W m^−2^). The online software ATRAN^24^ is used to compute the transmissivity of the atmosphere from the ground in the zenith direction (*t*_*atmos*,*λ*_(*θ* = 0°)) over the wavelength range [2–22] *μ*m. The resulting high resolution transmissivity spectrum is smoothed to reduce data to 0.1 *μ*m spectral resolution (see Fig. 2). Dependence on polar angle of the transmissivity is given by *t*_*atmos*,*λ*_(*θ*) = *t*_*atmos*,*λ*_(*θ* = 0°)^1/*cosθ*^ as in^5,12,25^. The heat load (*Q*) is calculated by simply subtracting the reflected flux and the electrical power output.

1 $$Q(T)={q}_{Sun}-{q}_{refl}-P(T),$$

where for the sake of limiting the number of variables, it is assumed that the panel does not transmit any radiation or that the transmitted part is included in the reflection term. The only other assumption made is that emission of the cell by radiative recombination, or external electroluminescence, is omitted. This term is usually negligible, but will tend to get much larger as the quality of cells gets better, *i.e*. when it tends to operate in the radiative limit with efficient internal luminescence extraction^26^. The reflected radiation flux is the sum of those reflected in the sup-bandgap (*λ* < *λ*_*BG*_) and sub-bandgap (*λ* > *λ*_*BG*_) spectral intervals:

2 $${q}_{refl}={\int }_{0.28\mu {\rm{m}}}^{{\lambda }_{BG}}{R}_{supBG}(\lambda )\,{q}_{Sun}(\lambda )\,d\lambda +{\int }_{{\lambda }_{BG}}^{4\mu {\rm{m}}}{R}_{subBG}(\lambda )\,{q}_{Sun}(\lambda )\,d\lambda $$

where *R*_*supBG*_(*λ*) and *R*_*subBG*_(*λ*) are the panel reflectance in the sup-bandgap and sub-bandgap intervals, respectively. Dependence on temperature of bandgap and any panel radiative property, would require data which are rather difficult to gather, hence is not included.

The heat source depends on temperature because electrical power output does:

3 $$P(T)={P}_{STC}[1+{\beta }_{P}(T-{T}_{STC})],$$

where *β*_*P*_ is the temperature coefficient of electrical power output and *T*_*STC*_ = 25 °C Heat is exchanged by the panel with the external environment by means of conduction/convection (simply noted *q*_*conv*_) and radiation (*q*_*rad*_),

4 $${q}_{conv}(T)=h(T-{T}_{\infty })$$

5 $${q}_{rad}(T)=2\pi {\int }_{2\mu {\rm{m}}}^{22\mu {\rm{m}}}\,{I}_{b,\lambda }(T)\,{\int }_{0}^{\pi \mathrm{/2}}\,{F}_{\lambda }(\theta ){t}_{atmos,\lambda }(\theta )\cos \,\theta \,\sin \,\theta \,d\theta d\lambda $$

where function *F*_*λ*_(*θ*) is the panel emittance, equal to (1 − *R*_*subBG*_(*λ*)) and *E*_*λ*_(*θ*) respectively on intervals [2–4] and [4–22] *μ*m, and *I*_*b*,*λ*_(*T*) is the Planck function. The atmosphere is virtually opaque between 22 and 50 *μ*m, hence the upper limit for the integral. It is of utmost importance to note that the above expression of the cooling radiative heat flux omits a fraction of the net exchange between the panel and the atmosphere, and thus is a bit underestimated (a few dozen of watts at maximum). An alternative calculation of this net cooling radiative heat flux would require estimating the flux emitted by the panel up to around 50 *μ*m -instead of that transmitted by the clear sky atmosphere- and subtracting the flux emitted by the atmosphere towards the Earth (which is not a straightforward task as it depends on both atmospheric temperature and composition profiles, see e.g.^27^) and possibly by the surroundings, and absorbed by the panel.

By equating the heat load and heat exchanged with the environment, an analytic expression of the panel equilibrium temperature is found:

6 $$T=\frac{h{T}_{\infty }+{q}_{Sun}-{q}_{refl}-{q}_{rad}(T)-{P}_{STC}\mathrm{(1}-{\beta }_{P}{T}_{STC})}{h+{\beta }_{P}{P}_{STC}}$$

Since the radiation heat flux (*q*_*rad*_) depends on panel temperature via the Planck function in Eq. 5, temperature is resolved iteratively.

Possibility of filtering out high energy photons to mitigate the heat load resulting from thermalization^2^ is explored by imposing a maximum reflectance (*R*_*UV*_ = 1) for wavelengths comprised between 0.28 *μ*m and an upper limit (*λ*_*UV*_). Impact on electrical power output in STC is modeled by assuming that most of the loss comes from a current drop. A correction factor $1-{\int }_{0.28\mu {\rm{m}}}^{{\lambda }_{UV}}\,EQE(\lambda )\,{q}_{Sun}\,d\lambda /{\int }_{0.28\mu {\rm{m}}}^{{\lambda }_{BG}}\,EQE(\lambda )\,{q}_{Sun}\,d\lambda$ is applied to *P*_*STC*_ in Eq. 6, where *EQE*(*λ*) is the external quantum efficiency of the cells.

Calculations are made by varying outdoor temperature from −5 °C to 45 °C and the heat transfer coefficient (*h*) from 6 to 62 W m^−2^ K^−1^ in order to cover various climate conditions, according to the wind speed trends in the contiguous United States reported in^28^ and convection coefficient estimates for forced air over flat surfaces^29^. It is paramount to keep in mind that engineering can also help intensify conductive/convective heat exchanges. Other parameters related to the panel design depends on the selected technology. In the case of crystalline silicon cells, the temperature coefficient cannot be smaller than its intrinsic value, *i.e*. when radiative and Auger recombinations are accounted for, and equal to −0.238% K^−1^ ^2^. Unless specified otherwise, it is set to the commonly reported value of −0.45% K^−1^ ^2^. Radiative properties, *i.e*. panel sup-bandgap (${R}_{supBG}(\lambda ),\,0.28\mu {\rm{m}} < \lambda  < {\lambda }_{BG}$) and sub-bandgap (${R}_{subBG}(\lambda ),{\lambda }_{BG} < \lambda \mathrm{ < 4}\mu {\rm{m}}$) reflectances and emittance ($E(\lambda ),4\,\mu {\rm{m}} < \lambda  < 22\,\mu {\rm{m}}$), and electrical output power in STC (*P*_*STC*_) depend on panel design. Specific choices made to assess their impact on thermal losses are specified wherever necessary. All input and output data are available upon request.
